# Phospholipases and Membrane Curvature: What Is Happening at the Surface?

**DOI:** 10.3390/membranes13020190

**Published:** 2023-02-03

**Authors:** María Laura Fanani, Ernesto Esteban Ambroggio

**Affiliations:** 1 Departamento de Química Biológica Ranwel Caputto, Facultad de Ciencias Químicas, Universidad Nacional de Córdoba, Córdoba X5000HUA, Argentina; 2 Centro de Investigaciones en Química Biológica de Córdoba (CIQUIBIC), CONICET, Haya de la Torre y Medina Allende, Ciudad Universitaria, Córdoba X5000HUA, Argentina

**Keywords:** sphingomyelinase, diacylglycerol, ceramide, giant unilamelar vesicles, curvature stress, amphiphilic drugs

## Abstract

In this revision work, we emphasize the close relationship between the action of phospholipases and the modulation of membrane curvature and curvature stress resulting from this activity. The alteration of the tridimensional structure of membranes upon the action of phospholipases is analyzed based on studies on model lipid membranes. The transient unbalance of both compositional and physical membrane properties between the hemilayers upon phospholipase activity lead to curvature tension and the catalysis of several membrane-related processes. Several proteins’ membrane-bound and soluble forms are susceptible to regulation by the curvature stress induced by phospholipase action, which has important consequences in cell signaling. Additionally, the modulation of membrane fusion by phospholipase products regulates membrane dynamics in several cellular scenarios. We commented on vesicle fusion in the Golgi-endoplasmic system, synaptic vesicle fusion to the plasma membrane, viral membrane fusion to host cell plasma membrane and gametes membrane fusion upon acrosomal reaction. Furthermore, we explored the modulation of membrane fusion by the asymmetric adsorption of amphiphilic drugs. A deep understanding of the relevance of lipid membrane structure, particularly membrane curvature and curvature stress, on different cellular events leads to the challenge of its regulation, which may become a powerful tool for pharmacological therapy.

## 1. Introduction

The cell membranes are active and dynamic places. They perceive the approach of external actors such as proteins, lipids, signal mediators, ions, neurotransmitters, hormones, etc. Several vital cellular events require remodeling the shape of the membrane in different ways [1]. These processes can be triggered by binding those effectors per-se or by changes in membrane composition linked to phospholipid metabolism. Phospholipases are responsible for altering the lipidic membrane composition in a fast and far–from–equilibrium way. Those changes, even when short-living, have strong consequences both in the bi-dimensional (lateral) [2,3] as well as the tridimensional membrane structure [4,5].

The concept that the action of phospholipases alters the physical environment of the membrane has been well established at the beginning of the century [6,7]. Those enzymes, in turn, are sensitive to the physical changes induced by the accumulation of their lipid products as well as of non-product or substrate lipids. In this way, they indirectly transmit information to other metabolic pathways through structural and electrostatic changes. The relationship between lateral phase separation and domain formation with the activity of phospholipases gained much attention in light of the “lipid raft” theory [8]. This implies both the generation of laterally segregated domains by phospholipases product as well as the regulation of phospholipase activity by heterogeneous membranes [3,9,10,11,12,13].

On the other hand, an increasing amount of evidence placed modulation of membrane curvature as a mayor membrane structure alteration upon de accumulation of enzymatically generated lipid products [4,14,15,16,17]. Those alterations might be of extreme importance in the context of several cellular events, whose significance is still being brought to light. The present work is aimed to review the consequences of the alteration of the tridimensional structure of membranes upon the action of phospholipases in different cellular scenarios, based mainly on evidence from studies on model lipid membranes. 

## 2. The Basics: Molecular Shapes and Membrane Curvature

In the 1990s, in his book, “Intermolecular and surface forms”, Israelachvili condensated the previous bibliography and exposed a clear relationship between the molecular shape of membrane components and the membrane structure [18]. Self-assembled lipid structures obey well-established thermodynamic rules. The anisotropic forces interacting between different parts of the amphiphilic molecules command the aggregation type of such molecules. The hydrophobic part of the amphiphilic molecules undergoes attractive interactions, which sharply decay with an increase in the intermolecular distance. On the other hand, intermolecular repulsion at the polar head level is driven mainly by electrostatic and steric contributions [19]. 

**Figure 1:**
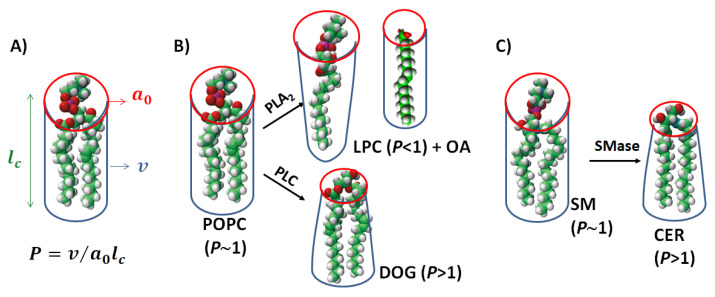
Schematic representation of lipid shapes of phospholipase substrates and products. (**A**) A description of packing parameter construction from the effective shapes of lipid molecules; (**B**) 1 2-dioleoyl-sn-glycero-3-phosphocholine (POPC) can be hydrolyzed by Phospholipase A_2_ (PLA_2_) to 1-oleoyl-2-hydroxy-sn-glycero-3-phosphocholine (LPC) and oleic acid (OA) or can be a substrate for Phospholipase C and be converted to 1-2-dioleoyl-sn-glycerol (DOG). (**C**) N-palmitoyl-D-erythro-sphingosylphosphorylcholine (SM) can be converted to N-palmitoyl-D-erythro-sphingosine (CER) by Sphingomyelinase (SMase). The figure highlights the molecular shapes of the lipids. POPC and SM have a large polar group and two acyl chains, giving a critical parameter (*P*) value close to 1 and forming planar bilayers. DOG and CER, which have small polar headgroups, are inverted-cone-shaped lipids with *P* > 1, promoting negative-curved structures. LPC has a large headgroup and a single acyl chain and is cone-shaped, showing *P* < 1 and stabilizing positive-curved structures. The molecular shape of OA will depend on its ionization state and surface electrostatic interaction. The 3D chemical structures source: https://avantilipids.com/products, accessed on 20 December 2022.

The resultant forces interactions define an optimal surface area per headgroup *a_0_*. Considering the latter parameter, the average volume of the hydrophobic portion of the molecule (*v*) and the maximum effective length that the hydrophobic chains can assume (the semiempirical parameter *l_c_*), the packing parameter $p=v/a_{0}l_{c}$ can be calculated (Figure 1). This parameter reflects the energetic constraint that the amphiphile undergoes when immersed in different self-assembled structures. Therefore, for reaching the minimum free energy, the amphiphiles with *p* smaller than 1/3 will form spherical micelles, non-spherical micelles when 1/3 < *p* < 1/2, bilayers when 1/2 < *p* < 1 and inverted structures when *p* > 1 [18]. Originally, the molecular geometric parameter was extracted from X-ray diffraction studies giving rise to semiempirical laws, which are useful for extrapolation to new amphiphiles of interest. Recently, a method for the determination of *p* based on molecular dynamics was reported, which has demonstrated high compliance with previously obtained *p* values [20].

Phosphatidylcholine (PC) and sphingomyelin (SM) are abundant in cell membranes. PC accounts for >50% of the phospholipids in most eukaryotic membranes and SM represents 25% of the phospholipids in the plasma membrane, being the most abundant sphingolipid in mammalian cells [21]. Those phospholipids are two-tailed amphiphiles with bulky phosphocholine at the polar head. This chemical structure confers an average cylindric geometry and therefore *P*~1 (Figure 1). For reference on the lipids abbreviations and chemical structures see Table 1.

Membranes enriched in PC or SM form lamellar structures of low curvature (infinite radius of curvature, *R_0_*), and are considered to have zero spontaneous curvature (*C_0_*) [22]. This indicates that the two lipid hemilayers are symmetric and have no tendency to form curved structures. It is important to notice that the *C_0_* of a particular lipid is defined as 1/*R_0_* of an unstressed aggregate of the pure lipid. However, the *C_0_* of a membrane is the result of the contributions of all the membrane components as well as the ions or factors interacting with its surface.

As a convention, conical molecules with a big headgroup and small hydrophobic portion show a tendency to form positively curved structures (with *R_0_* towards the hydrophobic portion) and an inverted conical molecule, with a small headgroup and large and bulky hydrophobic tails form negatively curved structures with the *R_0_* toward the water phase [22]. Phospholipases act on those phospholipid membranes in an asymmetric way giving rise to asymmetric lipid composition and, therefore, non-zero *C_0_* gathering curvature stress. If the cohesion of the bilayer cannot sustain the curvature stress, it will force non-lamellar structures to form.

The action of phospholipase A_2_ (PLA_2_) on PC produces a single-chained PC (lysoPC) and a free fatty acid (FFA). Depending on the length of the acyl chain, both components may remain as part of the membrane providing a different character to the membrane structure than the precursor PC. LysoPC has a bulky head group and a small hydrophobic portion, which results in an average cone shape and a *P*~0.4 [23]. On the other hand, the FFA can show different molecular shapes, depending on the acyl chain configuration and ionic state. As a relevant example, arachidonic acid (AA), a long polyunsaturated FFA (20:4), shows an inverted conical shape due to the bulky and dynamic conformation of the acyl chain (Table 1). 

Phospholipase C (PLC) cleaves PC at the headgroup level producing water-soluble phosphorylcholine and diacylglycerol (DAG). Similarly, sphingomyelinase (SMase) catalyzes the hydrolysis of SM into phosphocholine and ceramide (CER). The two-tailed lipid products show a very small hydrophilic headgroup and an inverted cone shape (see Figure 1 and [14]). The main difference between DAG and CER remains in the link between the hydrophilic group and the acyl chain. While DAG has a glycerol molecule as a backbone exposing to the interface a single OH group, CER presents an amide group linked to the C2 of the sphingosine backbone in addition to two OH groups (Table 1). This provides the possibility of forming intermolecular H-bonds between CER molecules and with other sphingolipids, which results in more compact lateral structures [24,25]. 

Finally, we will also comment on the action of phospholipase D (PLD), which hydrolyses PC to produce phosphatidic acid (PA) altering the electrostatic properties of the membrane. The acidic polar group of PA, even small in nature, induces a hydration sphere that occupies a considerably large hydrodynamic volume. However, the presence of counter ions, in particular the divalent ion calcium, would induce a surface compaction [26] and changes the overall lipid shape from cylindrical to an inverted cone. On the same trail, the signaling lipid phosphatidylinositol-4,5-bisphosphate (PIP_2_) has been described to be converted from an inverted-cone-shaped structure to a cone-shaped form in the presence of calcium [27].

Most of the above-mentioned lipid products are minority lipids in cell membranes. However, their enzymatic production is often localized in the inner leaflet of the plasma membrane and might reach high and transient local concentrations, which potentiate their effect on membrane structure and function [2,14].

## 3. On Membrane Curvature and Signal Transduction 

Since the 1990s, it has been clear that the action of enzymes involved in the production of signaling lipids affects horizontally the metabolic activity of plasma membrane through changes in the overall physical properties of the membrane, in particular through the membrane curvature [29,30]. In recent years, za systematic study demonstrated that the enzyme diacylglycerol kinase—whose substrate and products, DAG and PA, are signaling lipids—is allosterically modulated by membrane curvature [31,32]. The authors suggest that within a cell the enzyme works at a low basal level in locally flat membranes and its function is triggered by sites of highly curved membranes. 

Most of the enzymes mentioned in this work, including phospholipases, are cytosolic proteins, which require the binding, or a tight association, to the membrane for exerting their biological function. Bastiaens [33] proposed that the occurrence of curved membranes per-se affects the local concentration of amphitropic proteins and regulates the potency of molecular reactions at the membrane interface. Transient protein enrichment on surfaces of high convexity and depletion on surfaces of high concavity occurs due to local depletion of the protein cytosolic concentration in cavities, while the resupply is high in convex surfaces.

Once the enzymes become integrated into the membrane, the search for its ligand, namely the lipid subtract or other membrane-bound protein, becomes activated by a reduction of dimensionality, where the search of the ligand by lateral surface diffusion becomes more efficient than in the tridimensional system. An advanced study of such a system, where an amplified signal is released on the surface, mimicking the located generation of signaling lipids by a membrane-bound enzyme, has been recently performed [34]. Furthermore, the surface reaction of two given molecules has been demonstrated to be sensitive to membrane-induced local geometric perturbations, affecting the ligand concentration gradient [35].

When the cell membranes are constrained and remain in a low-curvature structure, the accumulation of phospholipase products may increase the structural frustration or curvature stress. Early reports demonstrated that the activity of CTP:phosphocholine cytidyltransferase, the core enzyme in the metabolic production of PC, as well as protein kinase C and other important enzymes in lipid signaling, are highly regulated by the membrane-stored curvature stress energy and respond to the presence of non-lamellar forming lipids [36,37,38,39,40]. Adding complexity to this system, the accumulation of oxidized lipid products in the membranes increases the ways of regulation of such metabolic and signaling pathways through alteration of the *C*_0_ and, therefore, of the membrane curvature stress energy [38,41]. 

**Figure 2:**
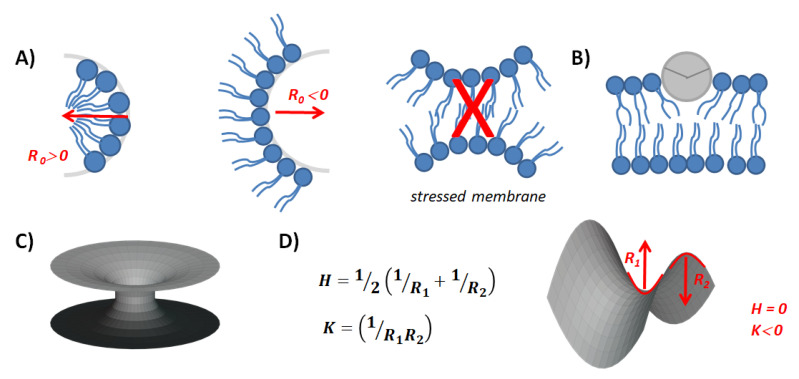
Illustration of curved lipid structures. (**A**) Positive- and negative-curved lipid surfaces. (**A**) membrane composed of negative curvature-forming lipids cannot be accommodated in a planar bilayer structure, which results in curvature stress. See also [22]. (**B**) The adsorption of an α-helix peptide to a membrane hemilayer induces a positive curvature bending and alters its *C_0_* and *K_B_*. The circle represents an axial view of an amphiphilic helix. Extracted from [42]. (**C**) Representation of an hourglass or stalk surface, which constitutes a fusion intermediate structure. (**D**) Saddle point surface with a zero main curvature (*H*) and a negative Gaussian curvature (*K*). The prolongation of this minimal surface results in a stalk. Extracted from [43].

Not only the accumulation of lipid products may alter the *C*_0_-inducing curvature stress, but the incorporation of cytosolic proteins and peptides into the membrane also might alter its geometrical properties. The adsorption of peptides, which organizes at the surface as amphipathic α-helixes has the potential to modulate the *C*_0_ of the membrane depending on the deepening of the helix into the hydrophobic portion of the membrane (Figure 2B). This depends on the polar angle, which separates the hydrophobic and hydrophilic sectors of the helix [42]. The modulation of *C*_0_ is due to the asymmetric perturbation of the two lipid leaflets of the bilayer. As a consequence, it also modifies the bending rigidity or stiffness of the membrane (*K_B_*), which denotes the energy per unit area required to produce a membrane curvature [22].

## 4. Membrane Curvature and the Recruitment of Protein Factors

Over the past decade, an increasing amount of evidence confirmed that specific proteins control the curvature of the membrane, whereas many others can sense the curvatures and bind to the specific geometrical cues [44]. Two molecular mechanisms have been described, where the adsorption of proteins modulates the curvature of membranes. The scaffolding mechanism, by which intrinsically curved proteins and oligomers induce large membrane curvature, takes place when the protein-membrane binding energy exceeds the *K_B_* and transfers their favorable association energy to induce the bending of the membranes. On the other hand, the molecular crowding mechanism proposes that crowding among protein molecules associated with the membrane surface at sufficiently high density generates lateral steric pressure, which provides an efficient force for the membrane deformation [45].

Furthermore, the binding of curvature-sensing proteins may be triggered by planar membranes which store membrane curvature stress. Several reviews are focused on how proteins may sense or induce membrane curvature. A recent review [44] is recommended for a general scenario of this subject. Here, we will emphasize how phospholipase activity plays a crucial role not only by affecting membrane curvature and packing but also allowing the recruitment of proteins to the lipid interface. 

**Figure 3:**
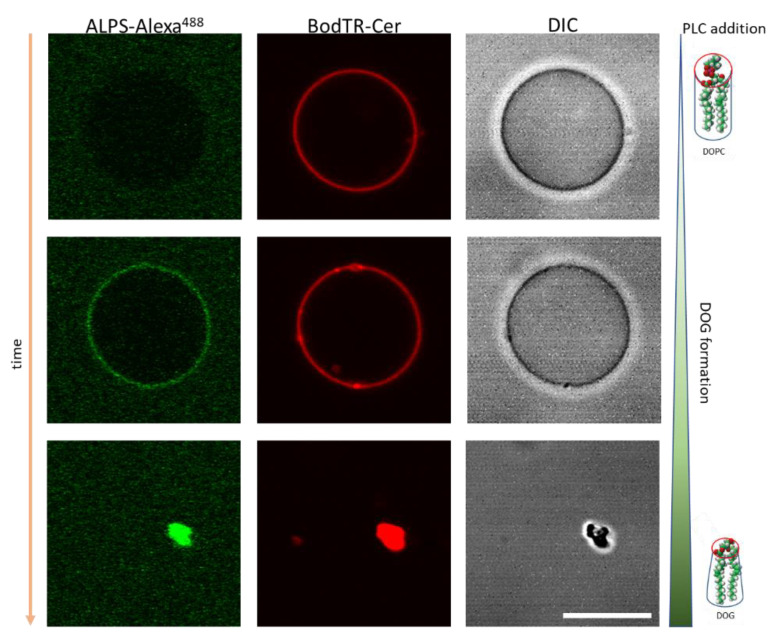
DOG generation by PLC on DOPC GUVs promotes ALPS binding. DOPC GUVs labeled with BodipyTR-ceramide (red channel) in the presence of ALPS-Alexa488 (green channel) and PLC. Scale bars 10 μm. Extracted from [46].

We demonstrated that the amphiphilic lipid-packing sensor motif of ArfGAP1 (ALPS) can bind the membrane of low total curvature membrane, such as giant unilamellar vesicles (GUVs) composed of DOPC, upon PLC, PLD and PLA_2_ activity [46]. When these enzymes start to hydrolyze the lipid substrate and to produce at the outer layer of the liposome products with high spontaneous curvature (DOG, DOPA and LysoPC, respectively) the ALPS motif now instead of binding high-curvature membranes (reported in [47]) associates to the membrane that retains a low total curvature but presents high curvature stress and lateral packing defects (Figure 3). 

Another example of phospholipase-mediated modulation of protein insertion is reported by Melero et al. [48] where they demonstrate the production of LysoPI due to the activity of Phospholipase B3 (PLB3, a type of PLA) on liposomes increases the binding of the COPII complex in a cellular environment. This effect is attributed to the change in *K_B_* upon de accumulation of the inverted-cone-shaped product of PLB3 action. The evidence of this mechanical facilitation of COPII budding by lysophospholipids evidenced a perfectly coordinated coupling between phospholipase action and vesical budding, spatially and temporally. 

Following that trend, a crucial role was attributed to phospholipase action on the membrane mechanical properties changes occurring during actin polymerization, which in turn induces the local recruitment of Arf1 and RAC1. This allows the binding of PIP5K1A whose presence is important for DAG production. In this way, the bar-domain containing protein Arfaptin 1 and 2 can also be recruited to the membrane to increase changes in lateral packing defects because of the augmentation of PLD and, therefore, the accumulation of PA lipids [49]. 

## 5. Membrane Fusion and Cell Function

The molecular mechanisms of membrane fusion are not completely understood at the present. One debated point is the relative importance of the roles of lipids and proteins in the fusion event. Once the mechanism of fusion in pure lipid systems is understood, the various ways by which proteins catalyze these processes in the cell may be identified [27,50]. However, it is primarily determined by the physics of lipid-lipid interactions.

For membranes to fuse, they first must come into close contact. This implies overcoming the electrostatic, steric and hydration effects between the opposing lipid membranes. This is followed by local bilayer destabilization, morphological deformations of the membrane and formation of non-bilayer intermediates [22,51]. The outer leaflets of the opposing membranes can mix forming a lipid stalk, characterized by an hourglass morphology (Figure 2C). 

This structure, described as a saddle point is present typically in cubic lipid phases and is characterized by showing two radii of curvature of opposite sign (Figure 2D). The main curvature of this structure (*H*) is the average of both radii and may cancel each other, thus giving the *H* value near zero. Therefore, its formation from a planar structure is not constrained by *K_B_*. However, an additional contribution to membrane deformation is described by the Gaussian curvature modulus *K_G_*. It reflects the energy involved in changes in the Gaussian curvature of membranes (*K*), which results from the product of the curvature radii of the membrane structure [43]. Since the *K_G_* of a saddle point structure is non-zero, the formation of the fusion pore implies a change in the Gaussian contribution to the elastic free energy [52]. 

Molecular dynamics simulations support, in general, the formation of the stalk pore as an intermediate of membrane fusion. The fusion process is triggered by a fluctuation in one of the monolayers, which results in some headgroups merging with the opposing monolayer. The stalk intermediate is stable only for a short time (∼10 ns), before being replaced by a hemifusion diaphragm in which the inner monolayers have merged. After 5 to 15 ns, a small fusion pore appears, the bilayer ruptures and the fusion process is complete. The speed of stalk formation and the opening of the fusion pore can be modulated by altering the lipid composition. The addition of phosphatidylethanolamine (PE), an inverted-cone-shaped lipid, accelerates the formation of the stalk intermediate, while vesicles containing LysoPC appear more resistant to the fusion [53].

Accordingly, a large amount of experimental evidence confirmed these observations and the role of inverted-cone-shaped lipids as promotors of the membrane fusion [54]. Furthermore, this phenomenon may be triggered by changes in the lipid membrane component shapes or electrostatic conditions, in a protein-independent way [50,55,56]. As mentioned above, the enzymatic generation of lipid products by phospholipases results in a rapid and asymmetric change in lipid composition. When lipid products are inverted-cone-shaped, it transiently exerts spontaneous curvature stress before their lateral diffusion or metabolization into structural lipids. This curvature energy can be released by catalyzing vesicle fusion, which is highly dependent on the enzyme catalytic rate [14,57]. 

The use of native cortical secretory vesicles as a biological model for studying membrane fusion has shown several parallelisms with simple model lipid membranes. Vesicle fusion in a cellular environment represents a much higher level of complexity than in lipid model membranes. In the biological scenario, it has been accepted that fusion proteins act as machines that use stored conformational energy to assemble closely apposed lipid bilayers. This leads to arrays of non-lamellar intermediate structures and fusion pore formation [54]. 

Fusion of the exocytic vesicular and plasma membranes is mediated by SNARE (soluble N-ethylmaleimide-sensitive factor attachment receptor) proteins triggered by the influx of calcium [27,58]. SNAREs are thought to catalyze the formation of a hemifusion transition state in which the proximal membrane leaflets have merged. Four SNARE motifs come together to form a four-helix parallel bundle known as the core complex, which is remarkably stable. All three SNARE types contain sequences that anchor them to the facing membranes through transmembrane segments, palmitoylation of the linker region and an extended hydrophobic α-helix section. Due to the parallel orientation of the SNARE motifs, SNARE assembly leads to the close apposition of the membranes. Then, the formation of the SNARE complex is an energy source that can be used to overcome barriers to fusion [58].

PLA_2_ metabolites LysoPC and AA regulated exocytosis in opposite directions. The exogenous addition of the negative-cone-shaped product, AA, increases catecholamine release from neurosecretory cells and has been shown to influence membrane fusion by enhancing vesicle docking. On the other hand, the conical product LysoPC inhibits 50% of exocytic fusion when present in only 1–2 mol% in the cell membrane. In the same trail, DAGs, a very active cone-shaped lipid produced by the action of PLC or DAG-kinases, are essential in the priming of the exocytosis [27,59,60].

The presence of small sphingolipids also influences cellular exocytosis. Sphingosine, a cationic intracellular signaling lipid, greatly increases the release of neurotransmitters in neuronal and neuroendocrine cells, whilst its zwitterionic analogue, sphingosine-1-phosphate (see Table 1) inhibited it at low concentrations [61]. Phospholipase anionic products, such as PA, PIP_2_ and other phosphoinositides as well as phosphatidylserine activates the vesicle exocytosis [27]. This evidences the importance of the electrostatic interaction of all the fusion machinery, and the important role of calcium ions. Both the anionic phospholipase product as well as the cationic lipid sphingosine regulates exocytosis by recruiting priming factors, which facilitate SNARE-catalyzed liposome fusion. 

Understanding fusion event as lipid or protein-driven phenomena is an oversimplification. Lipid composition is also proposed to modulate membrane fusion by changing membrane physical properties and altering the structure of virus fusion peptides [54]. One clear example is the Parvovirus Capsids where the capsid component VP1 protein presents a PLA_2_ activity. It has been shown that VP1 mutants alter viral infectivity and this effect is linked to the enzyme inefficacy in changing the lateral packing of the endosomal membranes [62]. Capsid mutations that reduce exposure of the PLA_2_ domain dramatically decrease infectivity, and mutations of residues around the fivefold axis of symmetry that are predicted to relax the pore can restore transduction activity in mutant particles lacking VP1 [63,64]. The viral PLA_2_ is not directly lytic but modifies membrane properties and may induce curvature by altering the lipid head groups to change their packing, likely causing endosomal escape. Only very transient or limited pore formation in the endosomal membrane must occur, as neither α-sarcin nor large dextrans enter the cytoplasm with incoming viral capsids [65,66].

In a different cellular scenario, membrane fusion is the protagonist of a vital event: the fusion of the sperm cell with the oocyte. The acrosome is a very large electron-dense granule covering about 50% of the nucleus in human sperm. Its secretion does not quite fit into any of the known modes of release. Rather, the acrosomal and sperm’s plasma membranes mix through a unique membrane fusion mode. Exocytosis of secretory vesicles causes the release of granules’ contents into the extracellular medium and the incorporation of the granules’ membranes into the plasma membrane. During sperm exocytosis, both the release of the acrosomal contents and changes in the plasma membrane are important. Agonist-receptor interactions promote calcium influx from the external medium into the cytosol, activating phospholipases. Enzymatic production of PIP_2_ at an early stage during sperm exocytosis initiates complex signaling cascades [67].

Sperm membranes have an unusual lipid composition. In addition to the phospholipids and sphingolipids present in all cell membranes, spermatozoa from many different species are rich in plasmalogens, which may elevate membrane fluidity or play a role in membrane fusion. Glycerophospholipids from rat spermatozoa have been known for decades to be rich in species containing long-chain polyunsaturated fatty acids (PUFA), especially 22:5n-6 and 20:4n-6. SM is also unique because it contains, N-linked to the sphingosine base, very-long-chain (VLC) PUFA of the n-6 series in the form of nonhydroxy VLCPUFA (n-V) and 2-hydroxy VLCPUFA (h-V), of which (n- and h-) 28:4n-6, 30:5n-6 and 32:5n-6 are the most abundant (Table 1 and [68]). Those species of SM containing VLC-PUFA (V-SM) are almost exclusively found in sperm heads whereas the sperm tails lack such SM species, their SM being instead rich in palmitic acid (PSM).

During acrosome reaction, a cholesterol efflux occurs with a decrease in cholesterol concentrations in sperm membranes. Concomitantly, an increase of CER containing VLCPUFA (V-CER) is detected, at expense of a decrease of the V-SM content due to almost complete hydrolysis of the head-located V-SM by SMase activity [68]. Thus, the enzymatic generation of CER leads to gametes that are relatively enriched in n-V, and especially in h-V CER. This prompts the question: what is the reason for such a specific location of these sphingolipids and its interconnection with the action of a sperm-associated SMase? 

Using model lipid membranes, we tested the fusion capacity of vesicles containing V-SM or canonic PSM after the action of SMase. The enzymatic production of V-CER was faster than that of PCER in similar conditions, and concomitantly, lipid mixing was more marked for the V-CER enriched vesicles. Furthermore, GUVs visualization indicated that the P-CER enrichment in the outer membrane leaflet of vesicles results in the formation of CER-enriched domains, as reported previously [2]. On the contrary, enzymatic production of V-CER results in a sudden increase of permeability and collapse of the bilayer structure [28]. This study provides direct evidence that the enzymatic generation of V-CER is an event that promotes dramatic changes in membrane structure, permeability and stability and that differ, in nature and extent, from those induced by the generation of the ubiquitous species PCER.

Atomistic molecular dynamics simulation of bilayers formed by V-SM, V-Cer of the n- and h- series and the corresponding V-SM/V-CER mixtures have been addressed to investigate the mechanism of this SMase-induced membrane restructuring [69]. The presence of V CER induced the V SM bilayer toward lower values of *K_G_*, being this behavior more noticeable in presence of n- than in the h-V CER. Furthermore, a decrease in the energy of curvature was found with the enrichment of V-CER in the bilayer. This trend evidences an increase in the bilayer instability with the V-CER enrichment, which can be associated with the membrane-related processes that take place during the acrosomal reaction and the fertilization process.

## 6. Pharmacologic Modulation of Membrane Curvature

A deep understanding of the relevance that lipid membrane structure and, particularly membrane curvature and curvature stress have on different cellular events, as those mentioned above, leads to the concept that its regulation may become a powerful tool for pharmacological therapy. Early on, Mouritsen highlighted the potentiality of pharmacological manipulation of cell membrane curvature using the nanomedicine [70]. He hypothesized that the membrane curvature stress might be modulated by specific molecular agents, such as fatty acids, Lysolipids, and other amphiphilic solutes, to construct intelligent drug-delivery systems that function by phospholipase activity triggering.

On this trend, amphiphilic drugs show a high potentiality to modulate membrane physical properties [71]. They comprise a large group of molecules with pharmacological functions that can naturally interact with the hydrophobic portion of cell membranes. This interaction determines their pharmacokinetic properties, such as their transport, biodistribution and accumulation, and hence, their efficacy [72]. Different from lipids that show very low solubility in the aqueous milieu, amphiphilic drugs usually show a critical micelle concentration in the micromolar range [71]. This indicates that monomers can reach a micromolar concentration before reaching equilibrium with the aggregated form of the drug. This provides a highly dynamic exchange of monomeric amphiphiles from the micellar structure to the aqueous medium and the membrane target. 

When amphiphilic drugs reach and are incorporated into the cell membrane, they produce a transient asymmetry of composition between both lipid hemilayers, as well as asymmetry in their physical properties. Dasgupta et al. [73] demonstrated that the asymmetric desorption of one membrane component, such as the ganglioside GM1, is translated to asymmetric *C*_0_ and, thus, to curvature stress. This can be relieved by the formation of internal nanotubes from originally spherical GUVs. Similarly, the asymmetric adsorption of an amphiphilic drug to the lipid bilayer might alter the balance between both hemilayers and modulate the *C*_0_ of cell membranes [71]. 

Dymond et al. [74] proposed that amphiphilic antineoplastic lipid analogues act through the reduction of membrane-curvature elastic stress. The alkylphospholipid analogues Miltefosine and Edelfosine were originally developed as antitumor agents in the 1980s. Although those drugs are no longer used in cancer therapy, Miltefosine is currently used as an antileishmanial agent and it is one of the few treatments that exist against the three types of leishmaniasis: cutaneous, mucosal and visceral. Its mechanism of action involves the disturbance of lipid-dependent cell signaling pathways [75]. Those alkylphospholipid analogues contain a phosphocholine headgroup and a single hydrophobic chain and show an inverted cone shape. The evaluation of the structure-activity relationship of alkyllysophospholipids from the literature provided a theoretical framework for the hypothesis that, when incorporated into the cell membrane the alkylphospholipid analogues disrupt membrane curvature elastic stress and inhibit membrane-associated protein activity, ultimately resulting in apoptosis [74].

Recently, we tested the capacity of Miltefosine to alter the highly curved membrane structures induced by the enzymatic production of DAG and CER, both inverted-cone-shaped phospholipase products with the capacity to disrupt lamellar lipid structures. The membrane insertion of the amphiphilic cone-shaped drug (with a low critical parameter *P* = 0.29) was expected to either directly modulate the membrane curvature or compensate for the generated stress by the inverted-cone-shaped lipids produced by PLC or SMase.

**Figure 4:**
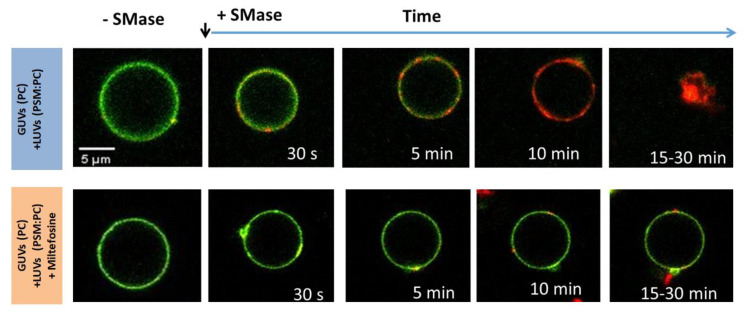
SMase promotes the fusion of GUVs with LUVs membranes and destabilization of the lipid bilayer upon CER accumulation. The presence of Miltefosine inhibits such a process. Confocal microscopy images of GUVs of PC labeled with NBD-PE (green) and fusogenic LUVs PSM:DOPE (80:20) or PSM:PE with 20 mol % of Miltefosine, labeled with Rho-PE (red). Samples were excited at the NBD excitation λ and images show the overlay of NBD and RHO emission channels. The red signal signs FRET occurring after vesicle fusion and lipid mixing. Extracted from [76].

The results of our work demonstrate that Miltefosine inhibits the vesicle fusion events, characteristic of the asymmetric enrichment of DAG or CER upon the enzymatic generation without affecting directly this activity [76]. Therefore, Miltefosine relaxed the curvature-tension of the enzymatically treated membranes and restricts bilayer destabilization, impairing vesicle fusion and the possibility that those signaling messengers diffuse laterally towards other vesicle membranes (Figure 4). Reinforcing the concept that this drug exerts its pharmacological function through the modulation of membrane properties, Miltefosine-resistant *Leishmania* promastigotes, as well as Miltefosine transiently treated parasites, show a modification in their membrane lipid profile [77]. This lipid regulation may have the capacity to compensate for the non-specific alteration of the intrinsic curvature of the membrane caused by Miltefosine.

## 7. Summary and Perspectives

In this revision work, we aimed to emphasize the close relationship between the action of phospholipases and the modulation of membrane curvature and curvature stress resulting from this activity. All the situations illustrated have in common the transient unbalance of both compositional and physical membrane properties between the hemilayers of the lipid bilayer upon phospholipase activity. This asymmetric production of lipids, whose local enrichment changes the *C_0_* of one of the hemilayers, leads to curvature tension and, therefore, its release by catalyzing several membrane-related processes. 

Even in the cases when the curvature stress induced by enzymatic production of lipids does not lead to membrane remodeling and the membrane remains in a planar conformation, the change in the membrane-stored curvature stress energy can regulate several cellular events. Either the membrane-bound or soluble forms of several proteins are susceptible to being regulated by the curvature stress induced by the phospholipase action [46]. This concept helps the understanding of the complex relationship between membrane dynamics and protein recruitment in cell signaling.

Of vital relevance is the modulation of membrane fusion by phospholipase products involved in several cellular scenarios. In the present work, we commented on vesicle fusion in the Golgi-endoplasmic system, synaptic vesicle fusion to the plasma membrane, viral membrane fusion to host cell plasma membrane and gametes membrane fusion upon acrosomal reaction. Furthermore, we explored the modulation of membrane fusion by the asymmetric adsorption of amphiphilic drugs [76]. This represents an underestimated mechanism of pharmacological action. Other amphiphilic drugs have been reported to act on the efficiency of vesicle fusion [61]. However, the pharmacological regulation of membrane curvature properties has seldom been approached and represents a new frontier for the design of new therapies.

## Figures and Tables

**Table 1:** Abbreviations in alphabetic order, names and chemical structures of the lipids are commented on in this work. Additionally, information on their relationship with the phospholipases commented on is given.

Lipid Abbreviation and Name) *	Chemical Structure **	Comments
CER; ceramide	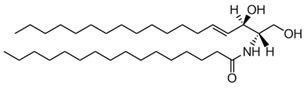 The structure corresponds to PCER (N-hexadecanoyl-D-erythro-sphingosine)	Product of SMase.
DAG, diacylglycerol	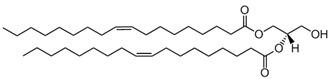 The structure corresponds to DOG (1-2-dioleoyl-sn-glycerol)	Product of PLC.
FFA; free fatty acid	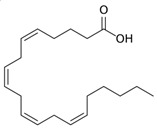 The structure corresponds to arachidonic acid (AA) or 5,8,11,14-all-cis-Eicosatetraenoic acid	Product of PLA_2_.
LysoPC; lysophosphatidylcholine	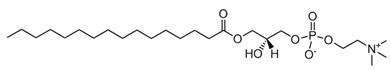 The structure corresponds to 2-oleoyl-sn-glycero-3-phosphocholine	Product of PLA_2_.
PA; phosphatidic acid	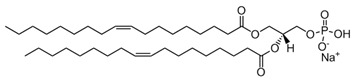 The structure corresponds to DOPA (1,2-dioleoyl-sn-glycero-3-phosphate, sodium salt)	Product of Phospholipase D (PLD).
PC; phosphatidylcholine	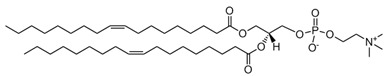 The structure corresponds to DOPC (1,2-dioleoyl-sn-glycero-3-phosphocholine)	Substrate of Phospholipase A_2_ (PLA_2_) and Phospholipase C (PLC).
PE; phosphatidylethanolamine	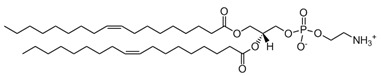 The structure corresponds to DOPE (1,2-dioleoyl-sn-glycero-3-phosphoethanolamine)	
PIP_2_; phosphatidylinositol-4,5-bisphosphate	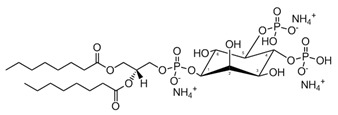 The structure corresponds to 1,2-dioctanoyl-sn-glycero-3-phospho-(1′-myo-inositol-4′,5′-bisphosphate) (ammonium salt)	Substrate of PLC.
SM; sphingomyelin	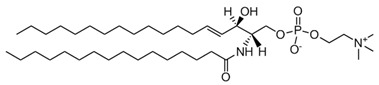 The structure corresponds to PSM (N-palmitoyl-D-erythro-sphingosylphosphorylcholine)	Substrate of Sphingomyelinase (SMase).
Sphingosine	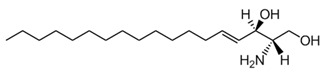 The structure corresponds to 2S, 3R(2S,3R,4E)-2-aminooctadec-4-ene-1,3-diol	
Sphingosine-1P	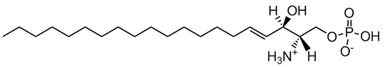 The structure corresponds to D-erythro-sphingosine-1-phosphate	
V-CER; CER that contain N-linked very long long-chain polyunsaturated fatty acids (VLCPUFA).	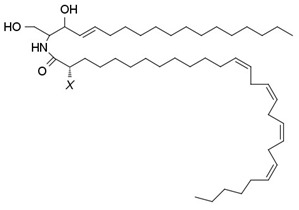 The structure corresponds to the CER containing an acyl chain C28:4. X = H or OH corresponding to n- or h- species, respectively.	Product of SMase
V-SM; SM that contains N-linked very long-chain polyunsaturated fatty acids (VLCPUFA)	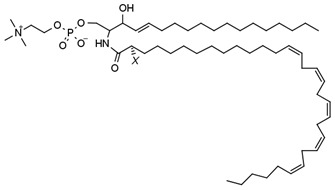 The structure corresponds to the SM containing an acyl chain C32:5. X = H or OH corresponding to n- or h- species, respectively.	Substrate of SMase

* The abbreviation will change when a specific acyl chain-containing lipid is referred to, as shown in the second column. ** Chemical structures source: https://avantilipids.com/products, accessed on 20 December 2022, except V-SM and V-CER, which are obtained from Ref. [28].

## Data Availability

Not applicable.
